# The Kurbo App: The Freemium Model and Developmental Behavior Concerns. Comment on “Impact of a Mobile App–Based Health Coaching and Behavior Change Program on Participant Engagement and Weight Status of Overweight and Obese Children: Retrospective Cohort Study”

**DOI:** 10.2196/17492

**Published:** 2021-02-25

**Authors:** Marcia Regina Vitolo

**Affiliations:** 1 Federal University of Health Sciences of Porto Alegre Porto Alegre Brazil

**Keywords:** childhood obesity, intervention, app

Recently, WW (the rebranded Weight Watchers) has launched the WW *Kurbo* app, which was designed to help overweight/obese children aged 8-17 years to lose weight with or without parental assistance if they are older than 13 years. A recent publication in this journal, authored by Cueto et al [1], is the first scientific study evaluating the impact of the *Kurbo* app on engagement and weight status among overweight children and adolescents. All the study data from children were provided by the *Kurbo* app and were obtained retrospectively. The analysis and conclusions, which should be considered with limitations, were done with users enrolled in coaching sessions, and the weight was self-reported. The impact on weight reduction was observed for those with more coaching sessions, which confirms that childhood obesity requires professional support. This app is available only upon payment. There is no information, so far, about its impact on obese children when they and their families downloaded the app for free and started to monitor the children’s food intake according to the traffic light diet.

The traffic light diet was first used as a part of a weight control program for overweight children aged 6-12 years with a multicomponent approach, published in 1980, including diet, exercise, and social learning principles [2]. On the other hand, there is no evidence that a traffic light system can be applied as an independent and effective tool in childhood obesity treatment. The traffic light system can be useful for the general population, labeling industry, and food education programs when the objective is to make people aware of food categories as in schools, cafeterias, and other public settings. However, food and nutrition knowledge is only the tip of the iceberg in the treatment of obesity in children. There is a large body of evidence that these children deal with emotional eating (eating in response to negative emotion or stress) [3], which makes treatment the biggest challenge of this century. The *Kurbo* app does not distinguish between the numerous factors related to childhood obesity such as etiology, overweight severity, gender, ethnicity, puberty stage, and self-esteem, and it is a dangerous oversimplification of obesity care and has a commercial interest. By providing a one-size-fits-all solution, it cannot provide the right approach to intervention, but more worrisomely it cannot control its side effects. The main criticism since it was launched is the potential risk for eating disorders since dieting is one of the most known predictors of their development [4]. Another risk of using this app is conflicts that will emerge in the child’s life; simply put, a child or teenager with great expectations of solving their overweight condition will struggle to have green scores while their neurotransmitters will push them to eat palatable foods (usually high in sugar and/or fat^.^) [5]. The frustration of facing red scores on the app screen can be an additional source of stress, reinforcing the need to eat tasty foods to compensate for negative feelings as a reward. Additionally, we should consider the risk of hiding, sneaking, or hoarding foods triggered by the embarrassment of eating in front of parents and others. Ignoring emotional outcomes, the *Kurbo* app design highlights the red score by displaying it in a larger size than green and yellow scores. 

It is a great paradox that this app has gone public, reaching millions of children and adolescents without being evaluated by randomized trials and emotional side-effects outcomes.
